# Are pre-analytical factors fully considered in forensic FFPE molecular analyses? A systematic review reveals the need for standardised procedures

**DOI:** 10.1007/s00414-025-03480-8

**Published:** 2025-04-02

**Authors:** Carlo Previderè, Serena Bonin, Calogero Cuttaia, Gianmarco Argentiero, Tommaso Livieri, Giovanni Cecchetto, Antonio Oliva, Paolo Fattorini

**Affiliations:** 1 Department of Public Health, Experimental and Forensic Medicine, Section of Legal Medicine and Forensic Sciences, University of Pavia, 27100 Pavia, Italy; 2 Department of Medical Sciences, University of Trieste, 34149 Trieste, Italy; 3 Department of Health Surveillance and Bioethics, Section of Legal Medicine, Fondazione Policlinico A. Gemelli IRCCS, Università Cattolica del Sacro Cuore, 00168 Rome, Italy

**Keywords:** Preanalytical, Molecular autopsy, FFPE, Standardization, Nucleic acids

## Abstract

The need for molecular analyses has become increasingly common in the forensic sciences, particularly in forensic pathology, to better shape the causes of death. This approach is called the “molecular autopsy,” where conventional medico-legal findings are often enhanced with specific molecular tests to provide reliable clinical and forensic diagnoses. In this context, FFPE (Formalin-Fixed Paraffin-Embedded) tissue samples collected during forensic autopsies are the only available specimens in retrospective studies for molecular DNA and/or RNA analyses. It is well known that pre-analytical parameters such as the agonal time, the PMI (Post-Mortem Interval), the fixation procedures, and the FFPE ageing and storage conditions can deeply impact the quality and quantity of the recovered nucleic acids, thus influencing the reliability of the downstream molecular tests. In the present study, we reviewed the recent forensic literature to establish whether these parameters are reported. Our survey showed that up to 34.9% and 40.5% of the 50 selected studies on DNA and RNA, respectively, reported the pre-analytical parameters mentioned above. Many publications did not report the length of agony (if any), which is an important parameter in RNA-based studies and estimations of the PMI; in addition, even relevant information on formalin tissue fixation procedures was often missing, thus impairing any critical evaluation of the PCR-based results. To address these issues, we propose the use of a simple form we set up to be filled out by Forensic Pathologists, where each pre-analytical step concerning the tissue samples collected during autopsy is accurately described and reported. In our opinion, this standardization will help the forensic community compare and evaluate the results of different molecular tests, thus increasing the reliability of the molecular results in forensics.

## Introduction

Formalin-fixed paraffin-embedded (FFPE) tissues are a cornerstone in diagnostic pathology, particularly for biopsies from living patients [1]. In this setting, stringent protocols are mandatory for microscopic and molecular analyses to ensure the integrity of the samples and the accuracy of the diagnoses (both histological and molecular), thus guaranteeing reliable and reproducible results. These protocols include careful handling and processing of the samples to prevent contamination, precise timing and conditions for fixation, and standardized methods for embedding and sectioning the biological tissues. Specific guidelines [1, 2] recommend the optimal fixation times to minimize cross-linking of nucleic acids, which can hinder their extraction and subsequent analysis. Standardized protocols for deparaffination and nucleic acid extraction should be set up and shared as this is crucial to obtain high-quality genetic material suitable for various downstream applications, most of which are based on Polymerase Chain Reaction (PCR) [3], Massive Parallel Sequencing (MPS) [4], and in situ hybridization [5].

FFPE tissues are also commonly used in forensic pathology, where tissue samples are collected during autopsies. These samples can be obtained during forensic or clinical autopsies, each with peculiar clinical and diagnostic purposes. These tissues are interesting for clinical diagnostics and integrating traditional autopsy findings with molecular data. This approach, often called *“*molecular autopsy*”* [6], aims to enhance the understanding of the causes of death by providing a molecular perspective. It is particularly valuable in cases of sudden unexpected deaths (SUD), often resulting from undiagnosed cardiac conditions (sudden cardiac death; SCD), which can be better understood through molecular autopsy by identifying genetic mutations associated with cardiac arrhythmias in approximately 20% of cases [7]. Conditions such as long QT syndrome, which may not leave any obvious anatomical markers, can be detected by analyzing genes involved in cardiac electrical activity [6, 7]. Additionally, molecular autopsy plays a crucial role in pharmacogenetic studies. By examining specific genes, such as those encoding cytochrome P450 enzymes [8], researchers can gain insights into how individuals metabolize drugs differently. Identifying these genetic variants can provide critical information for determining whether a drug played a role in the death and can also address future medical care for family members who may share the same genetic predispositions.

In Forensics, molecular analyses can also play a critical role in determining the cause of death in complex cases such as drownings or distinguishing hangings versus *post-mortem* suspensions. By assessing the vitality of lesions, forensic pathologists can differentiate between injuries sustained while the individual was still alive and those occurring *post-mortem* [9]. Therefore, to differentiate a hanging from a post-mortem suspension [10], molecular techniques can detect specific gene expression patterns or protein markers present only in *ante-mortem* injuries due to the body's response to trauma and hypoxia. These markers can provide the evidence that supports (or weakens) the presence of vital reactions at the time the injuries were sustained. In drowning cases, molecular studies can help identify biomarkers indicative of hypoxia [11] or water inhalation [12], which are signs of drowning while alive.

Moreover, molecular analyses can assist in estimating the *post-mortem* interval (PMI) [13], crucial in forensic investigations. The PMI represents the time elapsed since death, and can be challenging to determine accurately using conventional medico-legal approaches alone, especially when the body is found in advanced stages of decomposition. Molecular techniques, such as analyzing specific biochemical markers or gene expression profiles that change predictably over time after death [14–17], can provide more precise PMI estimates. These molecular indicators can offer valuable insights into the time frame of death, thereby aiding forensic pathologists in constructing a more accurate timeline of events surrounding the death. Additionally, molecular tests contribute significantly to epigenetic studies aimed at calculating the biological age of human remains, as they provide additional data points to narrow down the possible identity of the deceased. Epigenetic modifications, such as DNA methylation patterns, accumulate predictably in individual ageing. By examining these patterns, it is possible to estimate the biological age of an individual [18], which can sometimes differ from the chronological age due to environmental factors, lifestyle, or diseases. Another significant aspect of molecular analyses is the potential to identify individuals by comparing FFPE samples with biological specimens stored in pathology archives [19]. This approach is advantageous when dealing with unrecognizable human remains, where traditional identification methods such as visual recognition, fingerprints, or dental records may not be viable. By extracting and analyzing DNA from FFPE tissues, forensic scientists can match genetic profiles with those in existing DNA databases or biological samples collected from alleged relatives of the missing person. This approach not only aids in identifying unknown individuals but also helps resolve cases of missing persons. It can also be pivotal in criminal investigations where the deceased's identity is unknown [19].

Despite the potential of molecular analyses on FFPE samples, it is well-documented that the fixation and embedding processes can significantly damage nucleic acids [1, 2, 20–22]. Before considering these processes, however, several pre-analytical factors (such as agonal time, PMI, length of fixation, and the FFPE storage conditions) should be taken into account, as they can significantly influence the outcomes of molecular investigations due to enzymatic and/or spontaneous degradation of nucleic acids [23, 24]. In addition, modifications in the expression levels of non-coding RNAs such as miRNAs have been recorded at increasing *post-mortem* intervals [25], and other pre-analytical factors such as the agonal time can likely impact gene expression levels [14, 15, 24].

The aim of this study is to conduct a systematic review of scientific research involving RNA or DNA analyses in paraffin-embedded samples for forensic purposes (e.g., determining the cause of death, estimating *post-mortem* interval (PMI), or biological age). The review will assess whether the previously described pre-analytical factors have been adequately reported and considered. Based on the findings, the ultimate goal is to develop a standardized protocol that enables forensic pathologists to report all variables that could potentially impact the integrity and quality of the molecular analyses on FFPE samples.

## Materials and Methods

### Search strategy

**Fig. 1:**
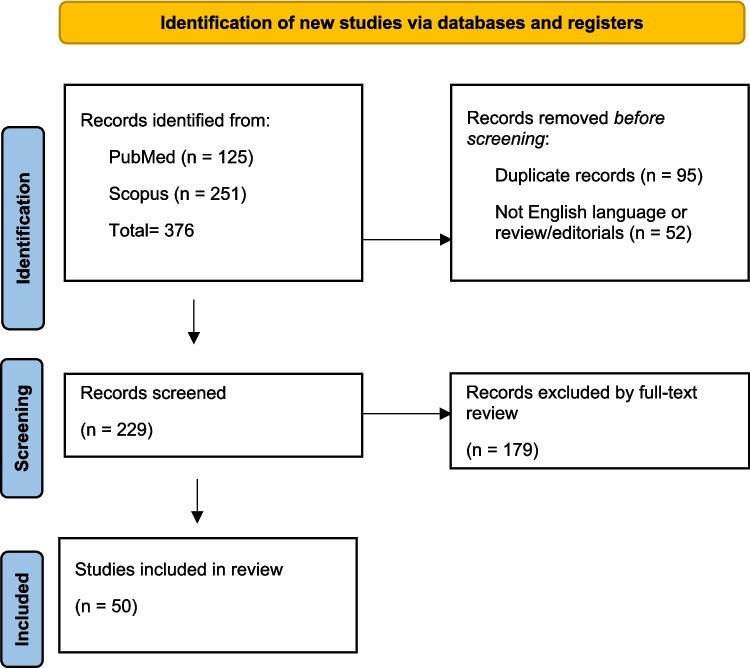
PRISMA 2020 Flow diagram illustrating the study selection process

A systematic literature review was carried out following the PRISMA (Preferred Reporting Items for Systematic Reviews and Meta-Analyses) 2020 criteria [26], using PubMed and Scopus as search engines. In the identification phase, all studies published between January 1, 2000, and December 31, 2023, were included. The keywords used for the search are listed in Table 1. At least one keyword for each field had to be present in the title or abstract of the studies. A total of 376 papers (125 from PubMed and 251 from Scopus, respectively) were initially considered (see Fig. 1). Of them, 95 duplicated records and 52 papers written in languages other than English or reviews/editorials were removed, with 229 original research papers reaching the screening phase.

**Table 1:** Keywords used for the search

FIELD 1	FIELD 2	FIELD 3
DNA and/or RNA and/or mRNA and/or miRNA	FFPE and/or formalin fixed paraffin embedded	autopsy and/or autoptic and/or forensic

Since this systematic review excluded a) studies on animals; b) studies on human tissues researching for non-human genetic material (e.g., viral or bacterial); c) studies on human tumors; d) studies on human tissues from biopsies, 179 papers were further removed. After the full-text review, 50 articles [6, 10, 27–74] were finally included and considered in the present study (see Fig. 1).

## Results and discussion

Out of the 50 selected papers, 27 focused on DNA (26 on genomic DNA [6, 28–32, 34, 35, 37, 40–43, 54, 56–58, 62–65, 68, 69, 71, 73, 74] and one on mitochondrial DNA [45]), 20 focused on RNA [10, 27, 33, 36, 38, 39, 44, 46–53, 59–61, 67, 72] and the remaining three considered both molecules [51, 66, 70].

**Fig. 2:**
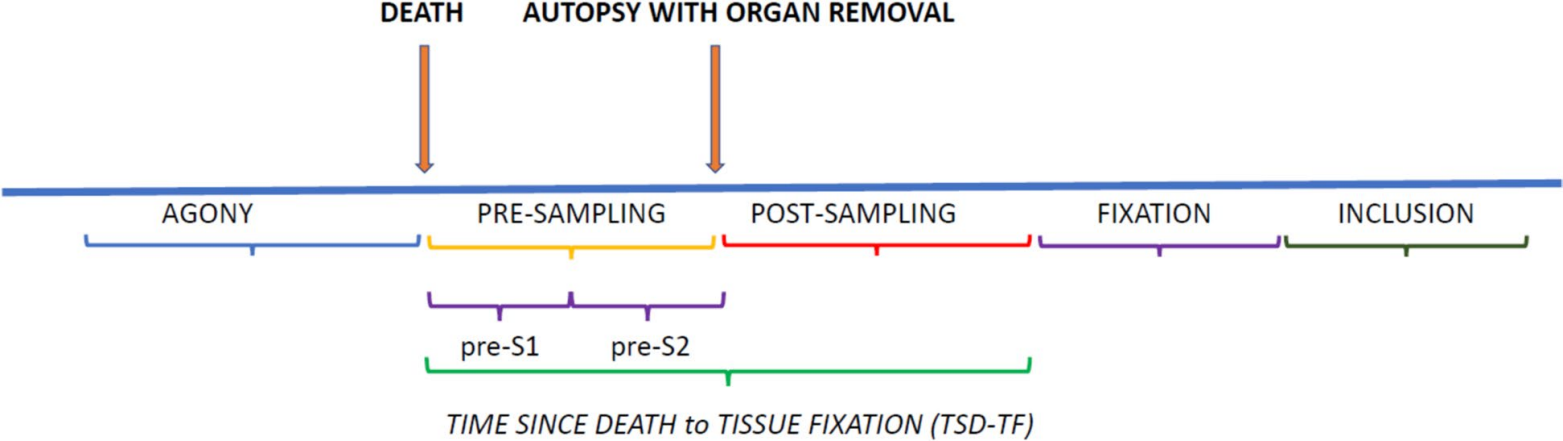
Phases of the pre-analytical process (before nucleic acid extraction) considered in the present review. See the text for a detailed description; *pre-S1* (from the time -or presumptive time- of the death to the morgue); *pre-S2* (the time elapsed in the morgue up to the organ removal during autopsy)

We initially considered both variables before the nucleic acid extraction step and those that characterize the molecular workflow (nucleic acids extraction and quantification, sample quality assessment, etc.). In agreement with shared models in Pathology and Molecular Pathology [1–3, 14, 15, 20–24], the pre-analytical phases of interest before nucleic acids’ extraction are shown in Fig. 2.

**Fig. 3:**
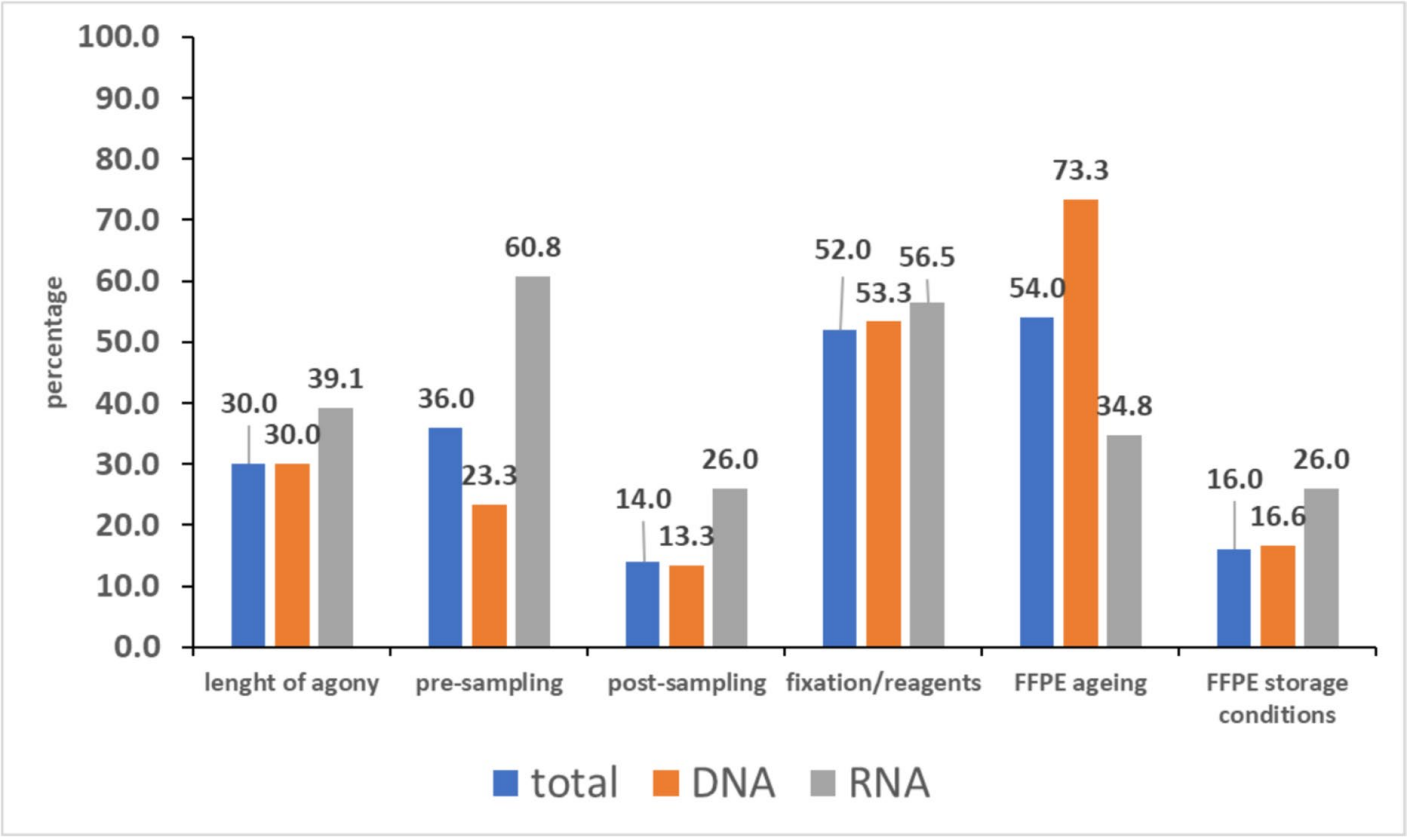
Pre-analytical factors (before nucleic acid extraction) considered in the 50 studies on DNA and/or RNA selected in this review. The columns indicate the percentage (y-axis) of studies on DNA and RNA, reporting the corresponding value (total: DNA plus RNA studies)

The main pre-analytical factors considered and their frequency among the studies analysed in this review are shown in Fig. 3. Since most forensic practitioners are responsible for managing only those phases, the following molecular workflow (DNA extraction and quantification, genetic profiling/sequencing) will not be considered in this review, although of great interest for a future assessment.

### Agony

Agony means, in medical terms, the terminal state of the body before death. Fifteen out of the 50 (30.0%) studies on DNA and/or RNA report the length of agony (LA), which is mainly referred to sudden unexplained deaths (SUD) [6, 28, 33, 57] or sudden cardiac deaths (SCD) [27, 29, 35, 46, 47, 67]; among them, 30.0% (9 out of 30) and 39.1% (9 out of 23) are studies on DNA or RNA, respectively, as shown in Fig. 3 (three studies were performed on both nucleic acids at the same time). These events are defined as non-violent, non-accidental, and unexpected deaths within 24 h from the onset of the symptoms [32], thus corresponding to a very short agonal time. A cause of death with very similar agony time is the one described in a case–control study by Courts [61], where FFPE tissues (heart and brain) from 14 sudden infant death syndrome (SIDS) patients were selected, and different RNA expression levels were observed for two miRNA in SIDS and controls. SIDS is defined as an unexplained death event of an infant < 1 year of age, with the onset of the fatal episode apparently occurring during sleep [75]. The amount and quality of the DNA extracted from myocardial samples collected from SIDS was assessed in the paper by van Deventer [40].

Vitošević et al. extracted DNA from FFPE samples of heart, liver, and brain tissues obtained from healthy individuals who had died suddenly due to violent causes. They evaluated the DNA yield, purity, and fragmentation using spectrophotometry and PCR amplification of the hTERT gene [31]. Longer agonal periods from 1 week to 504 h (21 days) are described by Boštjančič [48] and Bonin [59] in post myocardial infarction and fatal brain injured patients, respectively. Finally, mRNA expression was tested on FFPE lung tissue of a patient who died of a multiple organ failure and an agonal period corresponding to 16 days course of invasive mechanical ventilation [72].

While LA likely has no or negligible impact on the primary structure of DNA (and on the outcome of the following molecular analysis in FFPE tissue samples), several modifications can occur in gene expression levels during agony [14, 15, 24, 76, 77], depending on both the cause and the length of the agony itself. Therefore, it is essential to know not only the actual (or presumptive) cause of the agony but also accurate data on its lifespan, particularly for future RNA-based studies.

According to the GTEx (Genotype-Tissue Expression) Consortium [24] and the full recommendations posted for public use (http://biospecimens.cancer.gov/global/pdfs/caHUB_ANTWG_Postmortem_BPs.pdf), the following classification based on the 4-point Hardy Scale is recommended in forensics to define the LA properly:

1. Violent death (with a terminal phase estimated at < 10 min) such as in an accident, blunt force trauma, etc.;
2. Fast death of natural cause. SUDs of people who had been reasonably healthy, with a terminal phase estimated at < 1 h (e.g., sudden death from myocardial infarction);
3. Intermediate death after a terminal phase of 1–24 h, not classifiable as 2 or 4. The subject was ill, but death was unexpected;
4. Slow death after a long illness, with a terminal phase longer than 1 day (commonly cancer, chronic pulmonary disease); so, deaths that are not unexpected;

Since the length of agony cannot be accurately determined in most forensic casework, we suggest using “00” to point out this condition. Finally, any medical treatment (use of ventilator, mechanical support, or organ perfusion support) or medications administered during the agony period, as well as any clinical conditions (e.g. fever, hypotension, nutritional state) that may affect the quality of *post*-*mortem* research tissues should be reported.

### Time since death to tissue fixation (TSD-TF)

In Forensics, PMI (*post-mortem* interval) is the time that has elapsed from death to finding the body. Many research studies have tried to find an accurate and precise method to calculate the PMI, given that PMI can be more easily defined for early rather than late *post-mortem* intervals. Among the methods commonly used by pathologists, the time-related evaluation of the changes connected to body decomposition is among the most surveyed, even if biochemical [78] entomological [79], and lastly, molecular [80] approaches have been proposed over the past years to give support to the conventional forensic pathology methods [81]. As the estimation of the PMI is one of the crucial endpoints of the forensic practitioner, this term will be used in its conventional medico-legal meaning in this review, but pointing out that it does not correspond to the PMI as intended by the Genotype-Tissue Expression (GTEx) Consortium [24]. According to GTEx Consortium, in fact, PMI is the “total ischemic time for a donor”, which represents the time interval between the “actual death” and the “first tissue stabilization”.

In a forensic context, this time interval can be referred as the *time since death to tissue fixation* (TSD-TF), and its knowledge is particularly relevant for molecular studies, mainly for those dealing with gene expression [1, 2, 20–23, 62–66, 84]. In pathology, tissue stabilization is usually performed by fixation in 10% buffered formalin after placing the tissue section (approximately 5 mm thick) in a bio-cassette. Several steps, however, take place before stabilization (fixation) in forensics (see Fig. 2), and all of them should be carefully reported.

The data from the articles considered in the present review show that 18 out of the 50 (36.0%) selected studies on DNA and/or RNA include the time elapsed between death and autopsy with organ removal (or “pre-sampling” time according to Fig. 2); among them, 23.3% (7 out of 30) and 60.8% (14 out of 23) were studies on RNA and DNA, respectively (three researches were performed on both nucleic acids at the same time). This period of time ranged from 6 h to over 14 days for DNA studies, while it ranged from 1 h to 21 days for RNA studies. Most tissue samples collected for DNA studies showed a pre-sampling time of 6 to 48 h [31, 32, 41, 55, 65], while other tissues were collected for up to 14 days [32, 41, 66]. The knowledge of the length of this period is particularly relevant for studies dealing with gene expression [1, 2, 82, 83]. For this reason, most RNA papers report the pre-sampling time, which is from 1 to 48 h for a large proportion of the studies [36, 38, 44, 47–50, 55, 59, 60, 67]. Other articles account for pre-sampling times variable up to 21 days [33, 44, 49, 52, 55, 59].

Many modifications occur in nucleic acids after death. Besides the degradation of DNA and RNA molecules [23, 84], tissue-specific modifications related to expression patterns occur extensively. Furthermore, the trend of these changes (both increments and decrements of the gene expression) is time-dependent, at least within the first 29 h after death, in humans [14]. While the milestone study by Ferreira et al. [14] examined these changes for up to 29 h, it is likely that, in agreement with animal models [85], they persist for longer periods (up to 96 h).

Since the fixation procedure stabilizes the tissue structure by precipitating the cytoplasmatic substances, all that occurred before tissue fixation should be considered carefully and listed. Thus, from a practical point of view, TSD-TF should be composed of two phases (see Fig. 2): the first being the “pre-sampling” (pre-S) phase of the specimen, whereas the second one being the “post-sampling” (post-S) phase. Further, considering the common practice of delivering the body to the morgue and then storing it there before the autopsy for a few days, the pre-S phase has to be divided into the following two sub-phases: pre-S1 (from the time -or presumptive time- of the death to the morgue) and pre-S2 (the time elapsed in the morgue up to the organ removal during autopsy). Among the papers considered in the present review, only Kakimoto et al. [49] described that the bodies were left at room temperature for 3 to 24 h and then stored at 4 °C (before autopsy).

In forensic autopsies, a meticulous external body examination is required before evisceration. Moreover, a virtual autopsy conducted via CT scans has become routine practice, at least in some hospitals [86]. Therefore, it is clear that the exact time of “organ removal” does not precisely correspond to the beginning of the autoptic procedure. Given that both external examination and CT procedures can require a significant amount of time, often hours, it is essential to document their time and the temperatures at which they were conducted. Therefore, since a variable number of steps can compose the pre-S2 phase, each should be accurately reported (see Appendix).

Among the 50 reviewed papers, only 7 (14.0%) clearly state that the tissue was fixed “immediately” after the autoptic examination; out of them, no more than 13.3% (4 out of 30) and 26.0% (6 out of 23) were studies on DNA and RNA, respectively. In contrast, no data are available for the post-sampling phase of the remaining studies. The post-S phase can vary significantly in time and environmental conditions. In most cases, following macroscopic examination, organs are sliced into 5 mm thick sections and promptly fixed in buffered formalin within bio-cassettes [1, 2]. In such cases, the post-S is virtually “0” (zero), even if each procedure requires time (in addition, not all organs can be examined simultaneously). In many cases, however, this is not the rule. Occasionally, when bio-cassettes or formalin are unavailable, tissue samples are placed in an empty plastic container; afterwards, the tissue is sliced into thin sections and then fixed under ideal conditions at a later stage. When this occurs, reporting both time and temperature is recommended. In other cases, as the whole organ is required for instrumental examination, it is placed in formalin *in toto*. This is exemplified in studies on Sudden Cardiac Death (SCD) wherein the heart is analyzed via NMR [87], and only after NMR examination is the heart sliced into thin sections and finally fixed in formalin. Therefore, the fixation length *in toto* (along with temperature) should be reported, as well as other details on organ handling (see below).

### Fixation

In this review, fixation is the procedure by which the collected tissues (generally included in bio-cassettes) are completely submerged into formaldehyde solutions, with a recommended ratio between formaldehyde solutions and tissue of at least 10:1 (volume: volume; v:v) [88, 89]. Formalin is the aqueous solution of formaldehyde, with a maximum concentration of 40% (mass: volume) which corresponds to 37% v:v [88, 89].

The reviewed articles show that 26 out of 50 (52.0%) studies report the formalin concentration and/or how long the fixation lasted; among them 53.3% (16 out of 30) and 56.5% (13 out of 23) were studies on DNA and RNA, respectively (see Fig. 3). When provided, the buffered formaldehyde concentration ranged from 4 to 20%, with two studies reporting the use of un-buffered formalin [31, 63]. Most of the papers described the use of buffered 10% formalin for tissue fixation. Finally, paraformaldehyde was also used in two distinct studies [39, 47]. The fixation length ranged from 6 h to 31 years for DNA studies, whereas it ranged from 24 h to 37 months for RNA studies. The impact of formalin fixation on nucleic acid quality has been explored across a broad time range, from hours to 2 months [31], 2 to 136 days [29], and 3 to 31 years [64]. All the studies reported a progressive decrease of the nucleic acids yield and integrity, related to the length of formalin time fixation.

Few papers dealing with RNA reported a fixation time that was ≤ 48 h for most of the studies [47, 52, 66], but widely ranged from 1 week to 37 months [49], 2 days to 3 years [39], and from 12 to 20 months [36] in other studies. A single study divided the sample analyzed into two groups: < 48 h and > 48 h [59], without further specification. Vitošević [31] studied the DNA quality extracted from FFPE after fixation in 10% buffered and 4% un-buffered formalin for timeframes varying from 6 h to 2 months. Only a single paper [39] reported the fixation temperature (4 °C) in paraformaldehyde 4%, while for the rest of the papers, it is assumed that room temperature is the routine condition.

So, most studies reviewed here used samples fixed for extensive periods, well above the recommended standard of 24–48 h [1, 2, 90] or even 12–24 h, as indicated by ISO recommendations [89, 90]. Prolonged fixation increases the degradation/modification of the nucleic acids, making -in extreme conditions- the molecules unusable for any studies or, worse, leading to inaccurate conclusions, particularly when assessing gene expression levels [1, 2, 89–91]. In case of extensive degradation, where the tissue-specific reference genes have high threshold cycles (Ct > 40 cycles), gene expression analyses must not be performed [92].

It is also true, however, that small molecules (such as miRNA) seem to be more resistant to degradation when compared to longer ones (mRNA or DNA) [25]. Nonetheless, chemical modifications leading to the formation of mono-methylol adducts (hydroxymethyl derivatives) and dimers (crosslinks) occur even in small molecules [93], impairing future molecular analysis [93, 94].

In conclusion, the length of the treatment and the temperature should be reported in detail for each bio-cassette. When entire organs are fixed, special tissue handling (incisions of solid organs or openings of hollow organs) is usually performed to ensure adequate fixative penetration. In addition, as the recommended ratio of 10:1 between fixative and organ (v:v) is not easily feasible, the fixative solution shall be changed periodically [88, 89] before bio-cassette preparation. All these details should be specifically reported.

### Inclusion

After fixation, the specimen is usually dehydrated with alcohol solutions (another crucial step of the entire workflow) [95] and finally embedded (or included) in paraffin. The resulting FFPE samples can then be used for histological examination and stored for future analysis.

Twenty-seven out of the 50 (54.0%) selected studies reported the ageing of the FFPE; among them, 73.3% (22 out of 30) and 34.8% (8 out of 23) were studies on DNA and RNA, respectively (see Fig. 3). The ageing ranged from 30 days to 40 years for DNA studies, whereas it ranged from 7 days to 17 years for RNA studies. In addition, only 16.0% of the studies (8 out of 50) reported the environmental storage conditions. In detail, 16.6% (5 out 30) of the studies on DNA and 26.1% (6 out 23) of the studies on RNA stated that the FFPE were stored at “room temperature in the dark”.

Details on the ageing of the FFPE tissue and its environmental storage conditions (temperature, humidity, under vacuum, etc.) are also needed for a careful evaluation of the molecular results because several studies pointed out that FFPE samples undergo damage via oxidation/modification of the biomolecules [2, 20, 21, 23, 91, 95].

### Tissue

All RNA-based papers and all DNA studies, except one, reported the tissue collected for the molecular analyses. The tissue most targeted for DNA and RNA studies was the cardiac muscle followed by brain and liver tissues. The definition of the specific tissue samples selected for the study could be a relevant issue in evaluating the results of the molecular analyses, especially for studies assessing mRNA and miRNA gene expression. It is well known that *post-mortem* release of hydrolytic enzymes in the cell cytoplasm (autolysis) mainly affects soft tissues (pancreas, liver, spleen etc.) more than muscle tissues. These autolytic enzymes degrade the RNA in a tissue-specific way even if the impact of these modifications on the specific patterns of gene expression remains largely unknown [96].

### General considerations about the impact of the pre-analytical factors on FFPE samples molecular analyses

As molecular techniques become increasingly pivotal in forensic investigations, the need for meticulous documentation of the pre-analytical conditions becomes increasingly important and should represent an essential step of the entire workflow. This is particularly true for the “*omics*” approaches (genomics, transcriptomics, proteomics, and metabolomics), which aim to provide a comprehensive understanding of the biological samples by analyzing the entire set of genes, transcripts, proteins, and metabolites, respectively [15, 97–100]. So, the accuracy and reliability of these analyses heavily depend on the tissue samples’ quality, which, in turn, is influenced by the pre-analytical processes cited above.

Both DNA and RNA molecules undergo degradation after death [23, 84, 96]. Although tissue-specific degradation patterns are described for both molecules, the cause of death, the length of the *post-mortem* period, and environmental conditions, such as the temperature, are relevant factors that strongly influence the impact of such phenomena [23, 24, 84, 88, 89].

Besides quality modifications of the sample, even quantitative modifications can occur. For mRNA, changes in the expression pattern are well documented due to agonic factors [24, 76, 77, 88]. Always peculiar to mRNA, changes in the expression pattern in *post-mortem* time (at least in the first 29 h after death) are accurately described in a tissue-specific way [14–17, 76, 85, 88, 96]. However, it is still debated if *post-mortem* factors could interfere with epigenetic modifications of the DNA [89, 101].

Another important molecule to consider is microRNA (miRNA). MiRNAs are small, non-coding RNAs involved in the regulation of gene expression [25]. In the forensic context, miRNAs have garnered considerable interest due to their higher stability than other RNA molecules, including mRNAs. These features make miRNAs valuable biomarkers for *post-mortem* analyses aiming to identify the causes of death, define the PMI or determine the tissue origin of a sample [25, 102]. MiRNA, like any biomolecule, undergoes degradation and turnover [103] with mechanisms that are still not fully understood [104, 105]. However, it is clear that both physiological and pathological conditions can modulate the rate of their degradation [104, 105]. Therefore, the levels and integrity of miRNAs are expected to be influenced by various pre-analytical factors such as agony, the cause of death and all the phases before sample stabilization in formalin.

In addition, as a general rule for DNA and RNA (both mRNA and miRNA) molecules, prolonged fixation time increases the degradation and the chemical modification of the nucleic acids [1–3, 88–90, 93]. DNA, in general, is more stable than RNA in *post-mortem* tissues; however, the degradation rate of both nucleic acids also depends on environmental conditions and tissue type. Hard tissues like bone, teeth, and muscles preserve DNA and RNA better over extended periods, whereas soft tissues like the liver and brain degrade faster due to higher enzymatic activity and autolytic processes.

Similarly, long and inappropriate storage of the FFPE samples induces chemical damage to the molecules [1, 2, 20–22, 82, 92, 95]. Thus, both steps (tissue fixation and FFPE storage) are fundamental for critically evaluating the molecular data of the corresponding FFPE samples.

## Conclusions

In the present paper, we reviewed fifty papers where FFPE samples from forensic autopsies were considered for DNA and/or RNA-based tests. Our aim was to establish if pre-analytical parameters (or factors), which are fully considered in Molecular Pathology (such as agonal time, pre-fixation procedures, sample fixation methods, and paraffin embedding) [1, 2, 14, 15, 19, 20, 24, 76, 77, 82–85, 88–95] were also reported and fully considered in the forensic field. As shown in Fig. 3, most publications in forensics lack fundamental data regarding these pre-analytical parameters, which can influence the outcome of the molecular analyses and hinder an accurate interpretation of the results. It is for these reasons that both “sample description” and “sample storage conditions and duration (especially for FFPE samples)” are “essential” information in the MIQE (Minimal Information for the publication of Quantitative Real-Time PCR Experiment) checklist for authors, reviewers, and editors already since 2009 [82]. Similarly, the GTEx Consortium [14, 24] established stringent criteria for tissue sample collection because the length and cause of agony promote modification of gene expression patterns. RNA degradation, in fact, is a common finding in most samples collected from bodies with a PMI ≥ 8 h. Finally, even ISO recommendations accurately list each step of the sample workflow in the pre-analytical processes for RNA [88] and DNA [89] analyses, clearly stating what “shall”, “should”, “may”, or “can” be done to fulfil the requirements.

After having assessed the lack of information on the pre-analytical steps in most of the scientific papers considered in the present review, we have set up a comprehensive and standardized form (see Appendix) wherein the forensic pathologist should report every critical step in the pre-analytical processes by filling the corresponding field. The forensic examiner should be aware that the lack of these relevant data might cause misinterpretations and distortion of the molecular data used for a medico-legal diagnosis.

This simple and easy-to-fill form aims to encompass a detailed account of all variables that potentially impact the integrity and quality of molecular data, such as the tissue type collected during the autopsy, a detailed time-schedule of agonal factors, sample collection, handling, and period of storage, as well as the tissue fixation methods used. The standardization of the pre-analytical steps, according to the parameters described in the form, will allow the forensic community to compare and evaluate the results of the molecular approaches across different casework and laboratories. As a consequence, the reproducibility of the forensic studies will be improved as well as the reliability of the molecular data in legal context.

## Data Availability

The datasets generated during the current study are available from the corresponding author upon reasonable request.
